# A Self‐Powered Biochemical Sensor for Intelligent Agriculture Enabled by Signal Enhanced Triboelectric Nanogenerator

**DOI:** 10.1002/advs.202309824

**Published:** 2024-04-01

**Authors:** Along Gao, Qitao Zhou, Zhikang Cao, Wenxia Xu, Kang Zhou, Boyou Wang, Jing Pan, Caofeng Pan, Fan Xia

**Affiliations:** ^1^ State Key Laboratory of Biogeology and Environmental Geology Engineering Research Center of Nano‐Geomaterials of the Ministry of Education Faculty of Materials Science and Chemistry China University of Geosciences Wuhan 430074 China; ^2^ Beijing Institute of Nanoenergy and Nanosystems Chinese Academy of Sciences Beijing 100083 China

**Keywords:** intelligent agriculture, liquid‐solid interface, self‐powered biochemical sensor, triboelectric nanogenerator, volume effect

## Abstract

Precise agriculture based on intelligent agriculture plays a significant role in sustainable development. The agricultural Internet of Things (IoTs) is a crucial foundation for intelligent agriculture. However, the development of agricultural IoTs has led to exponential growth in various sensors, posing a major challenge in achieving long‐term stable power supply for these distributed sensors. Introducing a self‐powered active biochemical sensor can help, but current sensors have poor sensitivity and specificity making this application challenging. To overcome this limitation, a triboelectric nanogenerator (TENG)‐based self‐powered active urea sensor which demonstrates high sensitivity and specificity is developed. This device achieves signal enhancement by introducing a volume effect to enhance the utilization of charges through a novel dual‐electrode structure, and improves the specificity of urea detection by utilizing an enzyme‐catalyzed reaction. The device is successfully used to monitor the variation of urea concentration during crop growth with concentrations as low as 4 µm, without being significantly affected by common fertilizers such as potassium chloride or ammonium dihydrogen phosphate. This is the first self‐powered active biochemical sensor capable of highly specific and highly sensitive fertilizer detection, pointing toward a new direction for developing self‐powered active biochemical sensor systems within sustainable development‐oriented agricultural IoTs.

## Graphical abstract

A self‐powered urea sensor based on a triboelectric nanogenerator is demonstrated. This self‐powered urea sensor has achieved high sensitivity and high specificity detection of urea by introducing volume effect and enzyme‐catalyzed reaction.

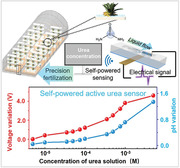

## 1 Introduction

Precision agriculture based on intelligent agriculture is crucial for sustainable development due to the high energy consumption and environmental consequences associated with fertilizer production and abuse.^[^ ^1^, ^2^, ^3^ ^]^ For instance, urea (CO(NH_2_)_2_), a key fertilizer that provides nutrients to more than half of the world's food crops, requires energy‐intensive processes and consumes roughly 2% of the world's total annual energy.^[^ ^4^, ^5^ ^]^ Therefore, accurate application of urea fertilization is paramount in mitigating both energy and environmental crises. The precise application of urea necessitates long‐term real‐time monitoring of its concentration. Traditional analytical techniques, such as inductively coupled plasma‐mass spectrometry (ICP‐MS), ICP‐optical emission spectroscopy (ICP‐OES), and flame atomic absorption spectrometry (FAAS), have been widely used for the multi‐elemental analysis of soils and fertilizers. However, these techniques generally involve long and complex sample preparation steps and the production of chemical waste, thus they do not respond efficiently to the demand for low‐cost and real‐time measurements in agriculture.^[^ ^6^ ^]^ The most important thing is that due to their size and energy consumption, they cannot be well integrated into the agricultural Internet of Things (IoTs) system for real‐time monitoring of urea. Therefore, it is necessary to incorporate corresponding sensors into the agricultural IoTs. Due to the wide distribution and large quantity of sensors in the agricultural IoTs, powering them through the power grid is not cost‐effective. On the other hand, relying on energy storage devices for power supply would require regular charging or replacement, as well as potential environmental concerns. Thus, the lack of green, efficient, low‐cost, distributed energy collection methods is a vital factor restricting the application of agricultural IoTs.

Building a self‐powered system based on various renewable energy technologies is expected to be an effective way to address the challenges mentioned above.^[^ ^7^, ^8^, ^9^, ^10^, ^11^, ^12^ ^]^ Among them, the triboelectric nanogenerator (TENG) is particularly remarkable. This is because TENG is gaining popularity as an effective power supply for agricultural IoTs.^[^ ^13^, ^14^ ^]^ Currently, TENG has achieved the conversion of randomly distributed, low‐frequency, irregular water (both raindrop and water‐flow energy) and wind energy in the agricultural environment into electrical energy, and for self‐powered sensing, nitrogen fixation, crop growth promotion, air and water purification in smart agriculture.^[^ ^15^, ^16^, ^17^, ^18^ ^]^ However, due to the relatively high energy consumption of spectrometers or biochemical sensors used for chemical sensing in soil or culture medium, it is currently not possible to rely on the power provided by TENG to achieve self‐powered sensing of fertilizer molecules in soil or culture medium.^[^ ^19^, ^20^ ^]^

Moreover, TENGs can be used as self‐powered active biochemical sensors due to their surface characteristics that react to external stimuli.^[^ ^21^, ^22^, ^23^, ^24^ ^]^ The advantage of using TENGs as both the power supply unit and sensing unit is a simplified system structure, making it advantageous for IoTs applications. However, existing TENG‐based self‐powered active biochemical sensors lack the required sensitivity and specificity to detect analytes, particularly in complex systems such as soil or culture solutions.^[^ ^25^ ^]^ Though modifying the sensors with proper probes is an effective way to improve specificity,^[^ ^26^ ^]^ there are limited methods for modifying friction materials of TENGs‐based sensors. Moreover, unfortunately, the modified friction material surface has more complex electrical properties, leading to instability in detection. A crucial challenge for sustainable agriculture is developing self‐powered sensors with high sensitivity and specificity for detecting urea concentration in soil or culture medium.

In this study, a TENG‐based self‐powered active biochemical sensor is developed, which can realize the accurate detection of urea with high sensitivity and specificity. To simplify signal capture, the device incorporates the volume effect^[^ ^27^ ^]^ through structural innovation, which significantly enhances the electrical output signal of TENG based on the solid–liquid interface. The sensor also utilizes urease‐catalyzed hydrolysis of urea to regulate the pH value,^[^ ^28^, ^29^ ^]^ enhancing the specificity of detection based on the pH‐sensitive TENG. Ultimately, this approach has been successful in monitoring the variation of urea concentration in the culture medium during plant growth.

## 2 Results and Discussion

**Figure 1:**
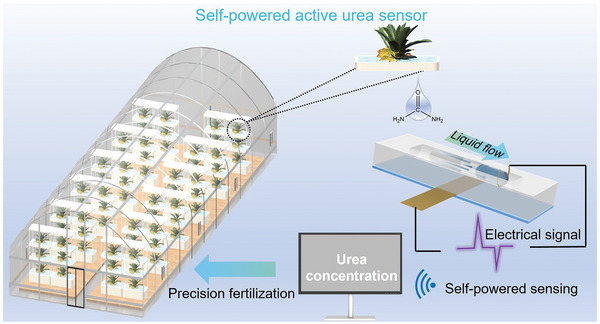
Working principle diagram of the self‐powered active biochemical sensing system for detecting urea concentration in plant culture medium by converting the mechanical energy of the liquid flow into electrical energy.

The as‐prepared sensor system is schematically shown in **Figure** **1**. The self‐powered biochemical sensor is composed of a glass slide, a fluid channel, and two electrodes including top and bottom electrodes, and the full dimensions of the device are shown in Figure S1 (Supporting Information). During the test, the device is fixed on the rotary mixer. The rotation of the device induces fluid movement back and forth. The flowing liquid can generate corresponding triboelectric electrical signals between the top and bottom electrodes. Movie S1 displays the real fluid motion in the fluid channel.

**Figure 2:**
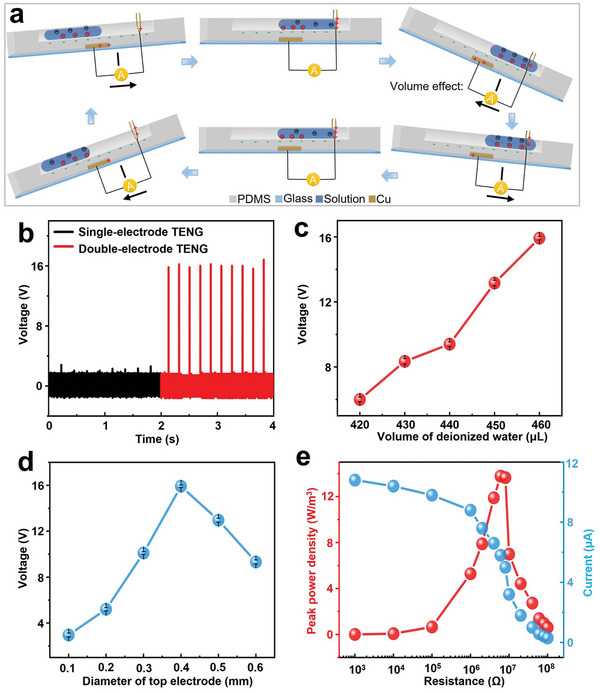
Analysis of the working mechanism and quantification of output performance. a) Working principle of TENG‐based self‐powered urea sensor with double electrode structure based on solid–liquid interface. b) The output voltage of the TENG‐based self‐powered sensor with and without the top electrode. c) The output voltages of the device as a function of the volume of water added. Values are means ± S.D. (five independent devices under the same conditions to obtain results). d) The output voltages of the device as a function of the volume of the diameter of top electrode. Values are means ± s.d. (5 independent devices under the same conditions to obtain results). e) Evolution of current peak and power peak with the increase of external resistance for the device with 460 µL of DI water.

To illustrate how the device achieves signal amplification through the volume effect, the working mechanism of the self‐powered biochemical sensor is demonstrated in **Figure** **2**a. According to the triboelectric series, PDMS is much more negative than water. As a result, when water flows over it, contact electrification occurs, causing an equal and opposite charge on both water and PDMS. At first, this device operates like a traditional one without a top electrode.^[^ ^30^ ^]^ However, once liquid contacts the top electrode and bridges it with the PDMS surface, the electrostatic induction effect takes place. This induces a spontaneous flow of positive charges from the bottom to the top electrode, resulting in an electric signal output between them. The incorporation of a top electrode transfers triboelectric charges based on bulk effect or volume effect, avoiding interfacial screening effects, and enabling the formation of high output voltage.^[^ ^31^ ^]^ The double‐electrode device shows a noteworthy open‐circuit voltage (*V*_oc_) of 16.2 V, while the device having only a bottom electrode has a *V*_oc_ less than 2.8 V, comparable only to our measurement's noise level (Figure 2b). This causes the corresponding short‐circuit current to increase from 5.6 to 26.4 µA (Figure S2a, Supporting Information). The transferred charge of devices also demonstrates similar trends (Figure S2b, Supporting Information). Consequently, these findings illustrate the successful introduction of the volume effect with the top electrode's design.

To further enhance the understanding of its operation principle, the device is analyzed from a circuit perspective and proposed the equivalent circuit model (Figure S3, Supporting Information). The fluid water is treated as a resistor and PDMS as a capacitor (*C_p_*), in which the water‐PDMS as top plate and the PDMS‐bottom electrode as bottom plate. At the interface between water and PDMS, an electric double layer (EDL) is established and regarded as *C*_1_. When the water contacts the top electrode, a new EDL is formed at the water‐top electrode interface (*C*_2_). *C*_1_, *C*_2_, and *C*_p_ are in series. Since the thickness of the EDL is negligible compared to that of the PDMS at the water‐solid interface, the whole equivalent capacitance *C* can be approximated as *C*_p_. According to equation: $U=\frac{Q}{C}=\frac{Qd}{\varepsilon A}$, where *ε* and *d* are the dielectric constant and the thickness of PDMS, respectively. The measured *A* and *Q* are 2.54 cm^2^ and 2.2 nC, respectively. Besides, the voltage is estimated to be 23.2 V which is close to the experimental measurement.

Then, the parameters of the device are quantitatively studied. When the rotate speed of rotary mixer is fixed, the diameter of top Cu wire electrode and volume of fluid will affect the output performance. Because they separately determine the area of the triboelectric material covered by the fluid, the area of contact between the fluid and electrodes, and the velocity of the fluid at the moment of contact. First, the influence of test fluid volume on the output performance of the device is investigated for optimization. Deionized (DI) water is chosen as the fluid in the microfluidic device. Figure 2c exhibits the output voltage as a function of volume of DI water for a fixed diameter of top Cu wire electrode of 0.4 mm. When the liquid volume is too small, gravity cannot overcome substrate viscosity, preventing sliding. With an increase in droplet size, both contact area and charge content increase, leading to an increase in output voltage. Nevertheless, unstable performance may result from liquid overflow during shaking if the droplet size is too large. Therefore, a fixed test droplet volume of 460 µL is utilized.

Then, for a fixed volume of DI water, the output performances of six devices with different diameters of Cu wire top electrodes are shown in Figure 2d. The peak values of *V*_oc_ display an upward trend from 3.2 to 16.2 V with an increasing diameter of top electrode from 0.1 to 0.4 mm. This phenomenon can be explained as follows, as the diameter of the top electrode increases, it improves the contact area between it and the DI water, resulting in enhanced output performance. However, if the diameter of the Cu wire electrode continues to increase, the output voltage signal decreases. This is due to an increased induction distance and reduced output signal caused by an increase in the thickness of PDMS on the bottom Cu foil electrode surface, which aligns with related reports.^[^ ^32^, ^33^, ^34^ ^]^ Therefore, there is a trade‐off between these two conflicting effects on output performance. The output current and peak power density are further investigated using different external loading resistances. It is evident that the current peak monotonically decreases as the loading resistance increases. For instance, when the load resistance rises from 1 to 100 MΩ, the output current peak decreases from 10.8 to 0.3 µA. The maximum instantaneous power density output of 13.78 W m^−3^ is achieved when the external loading resistance is set to 6 MΩ (shown in Figure 2e).

Moreover, the presence of free ions in the solution and the pH level of the liquid can influence both solid–liquid contact electrification and the formation of the subsequent EDL structure, ultimately leading to altered output performance. Thus, various aqueous solutions, such as NaCl, NaOH, and HCl, are subjected to testing using the device. First, the effect of NaCl concentration on the output performance of the device is investigated. From Figure S4 (Supporting Information), it can be seen that the peak values of *V*_oc_ increase gradually as the NaCl concentration increases to 1 mm. This is because the lower concentration of NaCl solution will act as a conductor with the increase of the conductivity, which is beneficial for electron transfer between water and PDMS.^[^ ^35^ ^]^ However, when the NaCl concentration continues to increase, the result of the screening effect caused by high‐concentration cations can be regarded as adjusting an EDL with a lower charge density. Thus, as depicted in Figure S4 (Supporting Information), the output voltage gradually decreases to 9.8 V with increasing NaCl concentration increases to 5 mm.

**Figure 3:**
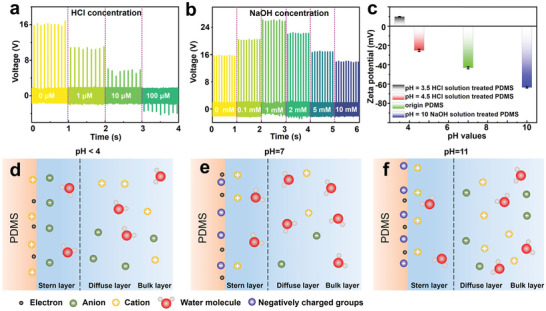
The influence of pH value on the output performance and surface charge state of the device. a) Output voltages of the self‐powered biochemical sensor, as a function of HCl concentration. b) Output voltages of the device, as a function of NaOH concentration. c) Zeta potentials of the as‐prepared PDMS surface and PDMS surface after being treated with different solutions. d) Schematic image of the solid–liquid interface when pH<isoelectric point (IEP). e) Schematic image of the solid–liquid interface when pH 7. f) Schematic image of the solid–liquid interface when pH>IEP.

Then, the study investigates the effect of different pH solutions on the performance of the device. As depicted in **Figure** **3**a, the output voltage of the device decreases as the concentration of HCl increases, with the triboelectric polarity reversing to −2 V at 100 µm. This reversal is possibly attributable to modifications in the surface state of PDMS induced by the pH of the solution, which transitions from above to below PDMS's isoelectric point,^[^ ^35^ ^]^ leading to the creation of a newly positively charged surface.^[^ ^31^ ^]^ When the concentration of NaOH is increased to 1 mm (Figure 3b), the use of NaOH solution results in a rise in output voltage from 16.2 to 25.8 V. It is postulated that exposing PDMS to NaOH solution augments the amount of polar Si─O bonds on its surface, enhancing the triboelectric charge.^[^ ^36^ ^]^ To test this hypothesis, the zeta potentials of PDMS surfaces have been measured at each stage, while maintaining a similar pH to the test solution. PDMS treated with NaOH solution at pH 10 has a more negative zeta potential than fresh PDMS (Figure 3c), corroborating the hypothesis. Conversely, PDMS exposed to HCl solution at pH 4.5 displays a less negative zeta potential than fresh PDMS, and when treated with HCl at pH 3.5, the PDMS surface exhibits a positive zeta potential. In order to gain a deeper understanding of the results mentioned above, it is necessary to identify the nature of the PDMS surface under different pH solutions after triboelectrification. To address this, EDL models have been proposed. The isoelectric point of a material determines which ions are preferentially adsorbed on its surface under different pH conditions. For PDMS, its isoelectric point is ≈pH 4.^[^ ^37^, ^38^ ^]^ When the pH value is less than 4, the surface adsorbs hydrogen ions due to the excessive hydrogen ions in the solution (Figure 3d), resulting in a positive zeta potential. Conversely, when the pH value is greater than 4, hydroxide ions are preferentially adsorbed on the surface, causing the zeta potential of the PDMS surface to become negative. In pure water, the solid surface is composed of two carriers – electrons separated and transferred at the interface and ions formed on the surface (as shown in Figure 3e).^[^ ^39^ ^]^ As the pH continues to rise and the solution becomes more alkaline, the increase of polar Si─O bonds will also improve the negative charges adsorption ability of the PDMS surface, resulting in a more negative zeta potential (Figure 3f).

**Figure 4:**
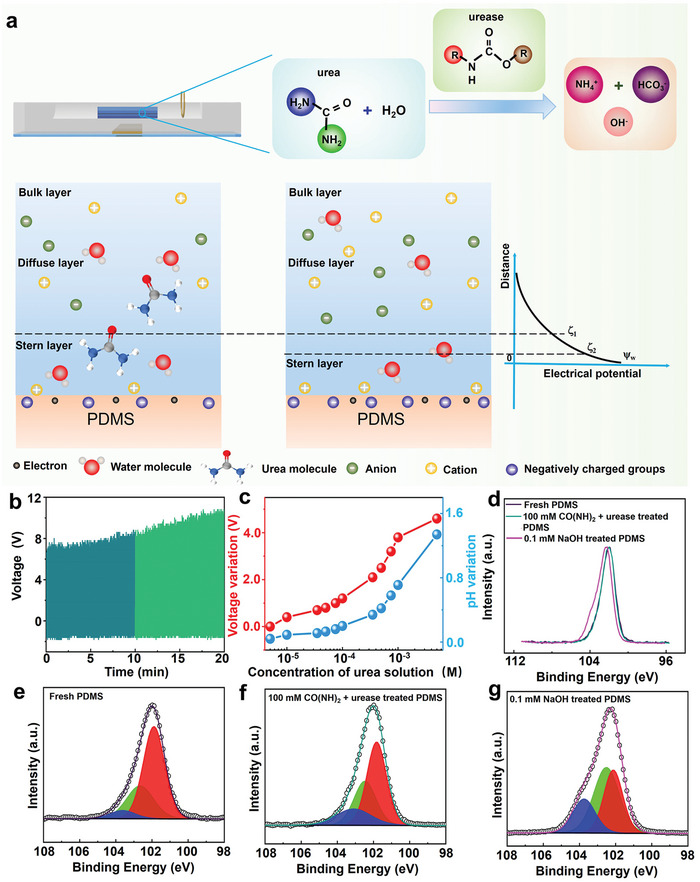
Characterization of surface property changes of triboelectric materials during testing. a) Schematic diagrams of the EDL between the solution and the PDMS surface of the sensor before and after the urease‐catalyzed reaction. b) Change of device output voltage signal after adding urease into the culture medium. c) The change of pH value of the solution and the increase of output voltage of the device after adding urease into urea solution with different concentrations. d–g) High‐resolution Si 2p X‐ray photoemission spectroscopy (XPS) peaks of fresh, CO(NH_2_)_2_ (100 mm) + urease, and NaOH (0.1 mm) ‐treated PDMS.

The results above have demonstrated that increasing the concentration of alkaline solution will enhance the output performance of the device. It is widely known that urea can be hydrolyzed into ammonium and hydroxyl ions (OH^−^) through the process of urease‐catalyzed reaction, leading to an increase in solution pH (**Figure** **4**a).^[^ ^29^ ^]^ To explain the formation of the new surface with a more negative zeta potential (*ζ*) during the catalytic reactions, the EDL model is further proposed. When the negatively charged PDMS surface is in contact with the reaction solution, positively charged components such as cations in the solution are attracted to the negatively charged PDMS surface, forming an EDL. Some of these cations combine strongly with PDMS to form a thin Stern layer. As these cations remain on the PDMS surface and are not transported with the fluid flow, they can be described as a new surface with a lower charge density of negative charges, which interacts with the solution. Therefore, the initial output voltage of the device with the catalytic reaction solution was lower than that with DI water. As the reaction proceeds, there are increasing numbers of hydroxide ions in the solution. The generated hydroxide ions will react with the PDMS surface, producing more negatively charged functional groups,^[^ ^40^ ^]^ resulting in a more negative *ζ* (i.e., *ζ_1_* > *ζ_2_*) and a corresponding increase in the output voltage of the device.

Therefore, the change of output performance of urea solution in the biochemical sensor before and after adding urease is investigated. Figure 4b,c illustrates the increase in pH value of urea solutions with varying concentrations after the addition of urease, as well as the corresponding increase in output voltage when tested in the device. This causes the corresponding short‐circuit current to increase from 10.8 to 18.2 µA (Figure S5a, Supporting Information). The transferred charge of the device also demonstrates similar trends (Figure S5b, Supporting Information). The voltage variation was determined by subtracting the output voltage of the device when the test solution was just added from the stable value of the device output voltage. It is evident that the voltage variation of the device is closely synchronized with the rise in pH value of the solution, showing a linear relationship in the two ranges of 0–100 and 100–1000 µm (Figure S6, Supporting Information). Moreover, the limit of detection (LOD) of CO(NH_2_)_2_ detection on the sensor is 1.2 µm.

In order to further clarify the surface changes of PDMS treated by different solutions, Kelvin probe force microscopy (KPFM), scanning electron microscope (SEM) and X‐ray photoelectron spectroscopy (XPS) analysis have been performed. To further clarify the potential changes and surface roughness of PDMS treated by different solutions, KPFM and SEM analysis of fresh and NaOH‐treated PDMS have been performed. The results clearly show that the surface potential of the PDMS surface increased after being treated with NaOH solution, which could enhance the ability of the PDMS surface to carry negative charges, thereby generating more frictional charges (Figure S7, Supporting Information).^[^ ^41^ ^]^The surface morphology of the resulting films is shown in Figure S8 (Supporting Information). No significant differences were not observed in the surface morphology and roughness of the fresh and NaOH‐treated PDMS films. To clarify the intriguing atomic ratio changes, high‐resolution XPS spectra of the Si 2p peak of fresh, NaOH, and urea + urease‐treated PDMS have been presented in Figure 4d. It can be seen that after NaOH treatment, the peak attributed to Si 2p drastically changes. However, the change of Si 2p peak corresponding to urea + urease treated PDMS surface was not obvious. Thus, based on the previous literature,^[^ ^36^, ^42^ ^]^ the peak has been deconvoluted into three components (Figure 4e–g): a peak at 102.1 eV corresponding to Si bonded to one O atom; a peak at 102.7 eV corresponding to Si bonded to two O atoms; and a peak at 103.7 eV corresponding to Si bonded to three or four O atoms. The third peak is associated with a highly oxidized surface with a silica‐like structure (SiO_x_, x = 3–4). The fresh PDMS surface consists of mainly Si─CH_3_ bonds with some Si─O and Si─OH bonds.^[^ ^43^ ^]^ NaOH treatment results in the increase of Si─O bonds at the expense of Si─OH bonds. Therefore, compared with the XPS peaks of the fresh PDMS surface, the intensity of the peaks at 102.7 and 103.7 eV increased, while the peak at 102.1 eV decreased correspondingly. This process may be similar to the following ionization reaction that occurs when the SiO_2_ surface contacts the alkaline solution:^[^ ^40^, ^44^, ^45^ ^]^

(1) $$\equiv \mathrm{Si}-\mathrm{OH}+OH^{-}\Leftrightarrow \equiv \mathrm{Si}-O^{-}+H_{2}O$$

Therefore, the amount of Si─O bonds increases at the expense of Si─OH bonds after alkaline treatment, which could increase the ability to carry negative charges of the PDMS surface, resulting in generating more triboelectric charge. The amount of OH^−^ produced after adding urease into the urea solution is limited, resulting in a less pronounced change in the three peaks compared to the sample treated with NaOH directly. However, the changes observed on the PDMS surface treated with urea + urease are sufficient to demonstrate their similarity.

**Figure 5:**
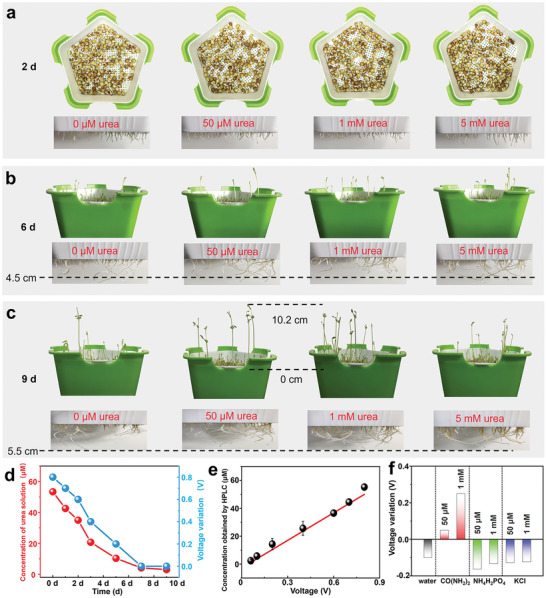
Performance of the self‐powered urea sensor. a–c) Photographs of stems and roots of beans cultured in different concentrations of urea solution. d) Voltage variation of the self‐powered urea sensor for analyzing culture medium with 50 µm urea after different incubation times. Validation of the self‐powered urea sensor for analyzing urea in culture medium using HPLC analysis. e) The correlation of the voltage variations from the self‐powered urea sensor and urea concentrations from the HPLC analysis. (*n* = 3 independent tests). f) Selectivity of the self‐powered urea sensor against various interfering substances.

The above results confirm that the addition of urease in urea solution can indeed enhance the signal output of biochemical sensors, demonstrating its potential as a urea sensor. As a demonstration, the germination of pea seeds in various concentrations of urea solution has been monitored. As shown in **Figure** **5**, we count the seedling heights and root lengths of pea seeds cultured in different concentrations of urea solution. After 2 days of germination, some seeds grow roots but do not germinate (Figure 5a). In comparison with the case where no urea was present, the growth of roots and stems is better in the culture medium infused with urea. In addition, significant‐difference analysis of physiological parameters in pea seeds cultivated on the 9th day, it can be inferred that an appropriate concentration of urea solution effectively promotes the growth of peas (Figure S9, Supporting Information). However, there is no significant difference in the growth state during the culture time with an escalating concentration of urea from 50 µm to 1 mm (Figure S9, Supporting Information). Since the nutrient solution needs regular replacement, using a 1 mm urea solution as the nutrient solution results in a copious quantity of unused urea after 10 days of cultivation, leading to waste.

The self‐powered biochemical sensors are used to monitor the nutrient solutions after different culture durations (Figure S10, Supporting Information; Figure 5d). At the same time, we evaluate the accuracy of the sensor for analyzing urea concentration in nutrient solutions using high‐performance liquid chromatography (HPLC). It can be observed from Figure 5d that the urea concentration measured by HPLC and the voltage variations recorded by the self‐powered biochemical sensor exhibit a similar trend with an increase in incubation time. Notably, even at low concentrations like 4 µm, the self‐powered biochemical sensor remains responsive to urea in the nutrient solution after a culture period of 7 days. However, the HPLC test reveals that the urea concentration decreases to ≈3 µm after 9 days of culture, whereas the device output voltage remains almost unchanged. Nevertheless, the detection sensitivity is sufficient for practical needs.^[^ ^46^ ^]^ The high correlation coefficient of 0.988 between urea concentration measured by HPLC and voltage variations measured by the self‐powered biochemical sensor (Figure 5e) indicates the potential of the self‐powered biochemical sensor as a urea concentration monitor for intelligent agriculture.

Moreover, specificity is a vital indicator for the device as a biochemical sensor. Despite the widely accepted specificity of the enzyme catalytic reaction, this study examined the device's selectivity by testing several salts commonly found in culture media (as shown in Figure 5f; Figure S11, Supporting Information). The findings suggest that the detection signal of DI water slightly decreases over time, which could be attributed to the reduction of water volume. On the other hand, when detecting potassium chloride or ammonium dihydrogen phosphate, the detection signal decreases rapidly due to cations in the salt solution adsorbing onto the PDMS surface, thus decreasing the number of negative surface charges. In contrast, only the urea solution shows a tendency to increase the output voltage during device operation after adding urease. In addition, to validate the detection of urea in a mixed solution, we measured the variation of the output voltage signal of the device after adding urease into a mixed solution containing 500 µm urea, 500 µm potassium chloride, and 500 µm potassium dihydrogen phosphate (Figure S12, Supporting Information). It can be observed that the output electrical signal of the mixed solution without urease does not increase over time, while the output electrical signal significantly increases with the presence of urease after its addition. The results showed that the device is capable of detecting urea even in a mixed solution, demonstrating its potential for accurately identifying specific analytes in challenging and diverse environments.

## 3 Conclusion

Herein, a TENG‐based self‐powered biochemical sensor for urea detection is demonstrated. Through innovative system design, the sensitivity and specificity are simultaneously improved. Specifically, the introduction of the volume effect enhances signal amplification and sensitivity, while the specific reaction of urea decomposition and solution pH enhancement catalyzed by urease generates a specific increase in the number of charges on the device surface, resulting in a specific detection signal. The device's capacity to monitor urea content in plant culture medium has been effectively demonstrated by test results. Therefore, it is anticipated that this device will be incorporated into the agricultural IoTs, enabling scientific and precise fertilization guidance.

## 4 Experimental Section

### Reagents and Materials

Polydimethylsiloxane (PDMS) prepolymer and curing agent were bought from Dow Corning. Conductive Cu wire with different diameters (0.1–0.6 mm) was purchased from Qinghe Shenghang Metal Material Company, China. Conductive Cu tapes with a thickness of 60 µm were purchased from Shenzhen Mileqi Company, China. Glass slide as a substrate with a length of 76.2 mm, width of 25.4 mm, and thickness of 1.1 mm was bought from Suzhou Shenying Optical Company, China. All the chemical reagents used in this experiment were from Sinopharm Chemical Reagents Company, and the chemicals were of analytical reagent grade and utilized without further purification. Deionized (DI) water was used throughout.

### Fabrication of the Device

PDMS elastomer (Sylgard 184, Dow Corning) was mixed with a curing agent at a mass ratio of 10:1 and degassed in a vacuum jar for 1 h to remove bubbles. A Cu foil tape with a width of 1 cm was adhered to the middle of the glass side as the bottom electrode and placed in a culture dish. Then, binding a Cu wire with a diameter of 0.4 mm on the acrylic cylinder with a length of 5 cm and a diameter of 1 cm. The acrylic cylinder with copper wire was placed in the middle of the glass side. Afterward, the PDMS slurry was poured into the culture dish and cured at 60 °C for 3 h, and the acrylic cylinder was peeled off from the PDMS. The Cu wire left on the PDMS is used as the top electrode.

### Sensing Process of the Device

To generate the electrical output signal for the sensing experiment, the device was fixed on the rotary mixer with an external power supply to make the fluid slide back and forth in the device at a certain frequency. Then, 460 µL of different fluids were dropped into the fluid channel of the device. Finally, owing to the contact electrification between the PDMS and liquid interface, their corresponding output signals were measured.

### The Cultivation of Plants

To control the experimental operating environment, an air conditioner and humidifier are used to maintain an environmental temperature of 25 °C and a relative humidity of 60%. In the experiment of germination promotion, the plant culture was performed in a darkroom. Chinese pea seeds were purchased online. To improve the germination rate and speed of pea seeds, they were soaked in urea solutions of different concentrations for 12 h before sowing. Pea seeds cultivated using a water‐soluble solution without the addition of urea were used as the control group. To improve the statistical efficiency of physiological parameters in pea seeds, 20 independent pea seeds were randomly selected from each parallel sample for analysis using a random number table method. The urea solution in the nursery pots is collected daily, and the concentration of urea is measured by self‐powered biochemical sensor and HPLC.

### Characterization

To test the TENG property, the output currents and voltages were performed by a low‐noise current preamplifier (SR570, Stanford Research Systems, Inc., Sunnyvale, CA, USA) and a digital phosphor oscilloscope (DPO 3052, Tektronix, Inc., Beaverton, OR, USA). The charge density from the output signals was measured using an electrometer (Keithley 6514, Cleveland, OH, USA). The zeta potential (*ζ*) of the PDMS surfaces at each stage of the catalytic reaction was determined using a SurPASS Electrokinetic Analyzer (Anton Paar GmbH, Austria). A background electrolyte of 1 mmol L^−1^ KCl solution was used during the experiments. X‐ray photoelectron spectroscopy (XPS) data were recorded using a Thermo Scientific K‐Alpha XPS system (Thermo Fisher Scientific, UK). High‐Performance Liquid Chromatography (HPLC) tests of urea concentration in nutrient solutions were done on Shimadzu LC‐20AT using an Agela Venusil AQ‐C18 column. The morphologies of the PDMS films were analyzed with a field‐emission scanning electron microscope (FE‐SEM) (S‐4800, Hitachi, Tokyo, Japan). A Bruker Dimension Icon scanning probe microscope mounted with a Pt/Ir coated conducting atomic force microscope (AFM) tip was used for all the Kelvin probe force microscopy (KPFM) measurements.

## Conflict of Interest

The authors declare no conflict of interest.

## Supporting information

Supporting Information

Supporting Information

## Data Availability

Research data are not shared.
