# Supplementary material for: A Self‐Powered Biochemical Sensor for Intelligent Agriculture Enabled by Signal Enhanced Triboelectric Nanogenerator
Source: Adv Sci (Weinh). 2024 Apr 1;11(22):2309824. doi: 10.1002/advs.202309824 (PMC11165538; doi:10.1002/advs.202309824)
Supplement: Supplementary file 1 — Supporting Information [file ADVS-11-2309824-s002.pdf]

ADVANCED SCIENCE
Open Access

Supporting Information

for *Adv. Sci.*, DOI 10.1002/advs.202309824

A Self-Powered Biochemical Sensor for Intelligent Agriculture Enabled by Signal Enhanced Triboelectric Nanogenerator

Along Gao, Qitao Zhou, Zhikang Cao, Wenxia Xu, Kang Zhou, Boyou Wang, Jing Pan, Caofeng Pan* and Fan Xia**

Supporting Information

A Self-Powered Biochemical Sensor for Intelligent Agriculture Enabled by Signal Enhanced Triboelectric Nanogenerator

Along Gao, Qitao Zhou, Zhikang Cao, Wenxia Xu, Kang Zhou, Boyou Wang, Jing Pan, Caofeng Pan*, Fan Xia**

A. Gao, Prof. Q. Zhou, Z. Cao, W. Xu, K. Zhou, B. Wang, Prof. J. Pan and Prof. F. Xia
State Key Laboratory of Biogeology and Environmental Geology, Engineering Research Center of Nano-Geomaterials of the Ministry of Education, Faculty of Materials Science and Chemistry, China University of Geosciences, Wuhan 430074, China.
E-mail: zhouqitao@cug.edu.cn; xiafan@cug.edu.cn

Prof. C. Pan
Beijing Institute of Nanoenergy and Nanosystems, Chinese Academy of Sciences, Beijing 100083, China.
E-mail: cfpan@binn.cas.cn;

Key words: Self-powered biochemical sensor; Intelligent agriculture; Triboelectric nanogenerator; Volume effect; Liquid-solid interface

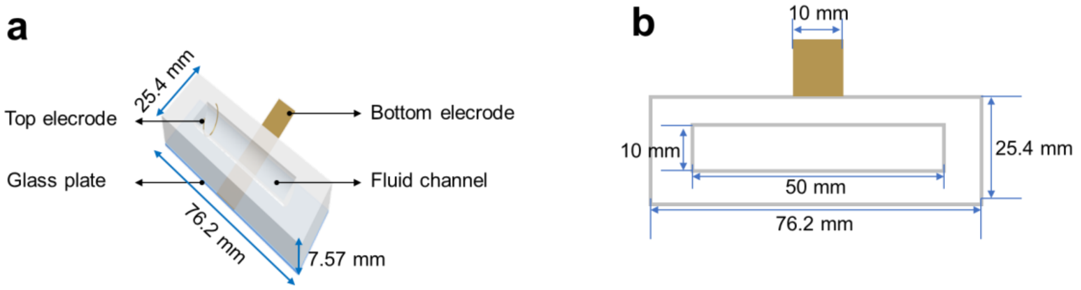

Figure S1. (a) The full dimensions the TENG-based self-powered urea sensor. (b) Dimensions of the device in top view.

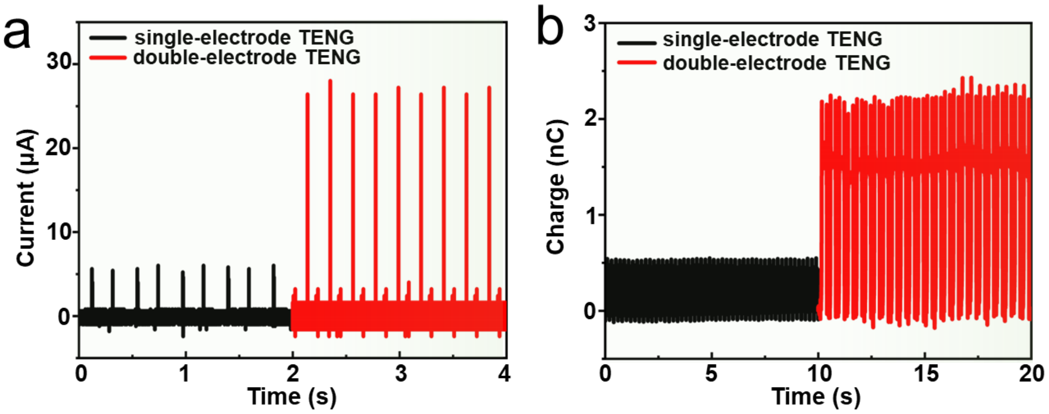

Figure S2. (a) The short-circuit current of the TENG-based self-powered sensor with and without the top electrode. (b) The transferred charge of the TENG-based self-powered sensor with and without the top electrode.

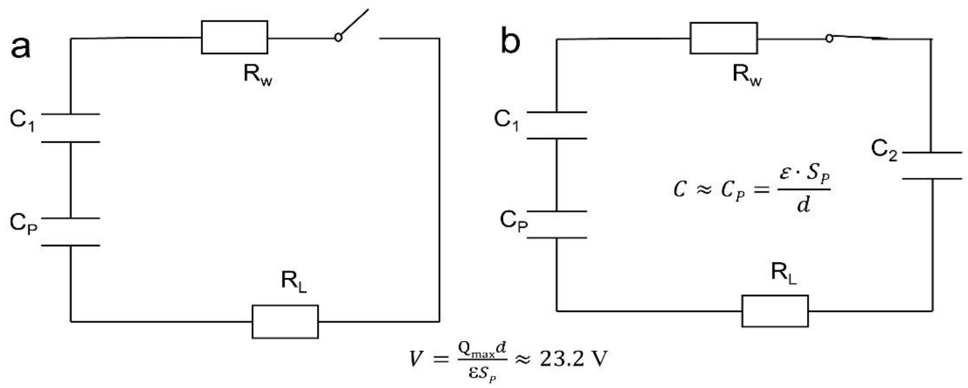

$$V = \frac{Q_{max} d}{\varepsilon S_P} \approx 23.2\ \mathrm{V}$$

Figure S3. Circuit model. (a) In the switched-off mode, there is no capacitor formed at the water/ top Cu electrode interface. As a result, C_P and C_1 remain in an open circuit and there is no charge flow between them. (b) When the top Cu electrode and PDMS are connected by the water droplet (switched-on mode), another capacitor, C_2, is established at the water/ top Cu electrode interface, forming a closed circuit.

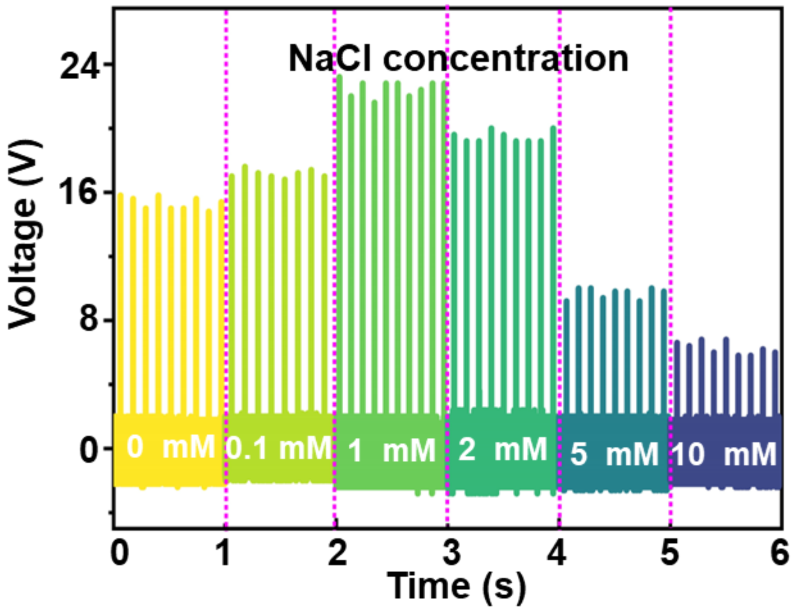

Figure S4. Output voltages of the self-powered biochemical sensor, as a function of NaCl concentration.

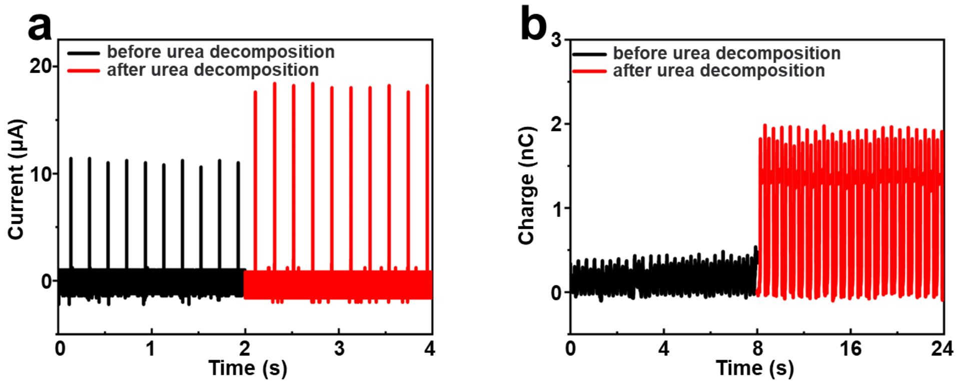

Figure S5. Change of device output (a) short-circuit current and (b) transferred charge signal after adding urease into the culture medium.

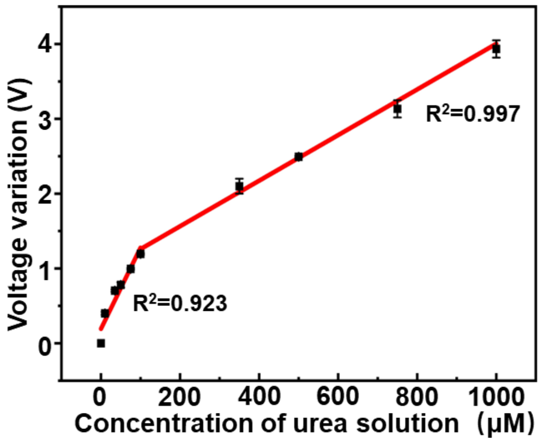

Figure S6. Calibration curve representing the relationship between the voltage variation and urea solution concentration. Values are means ± s.d. (n= 3 independent tests).

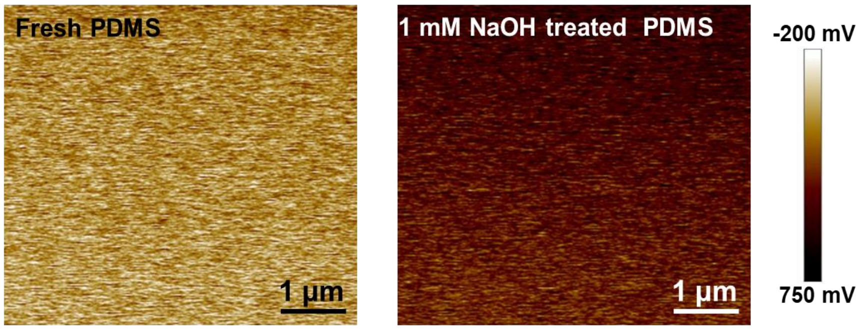

Figure S7. Surface charge potential images for the fresh and NaOH treated PDMS.

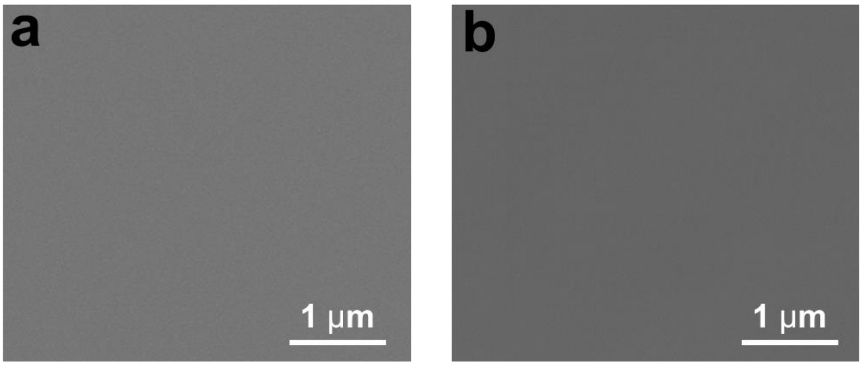

Figure S8. The SEM morphological of the (a) fresh and (b) NaOH treated PDMS.

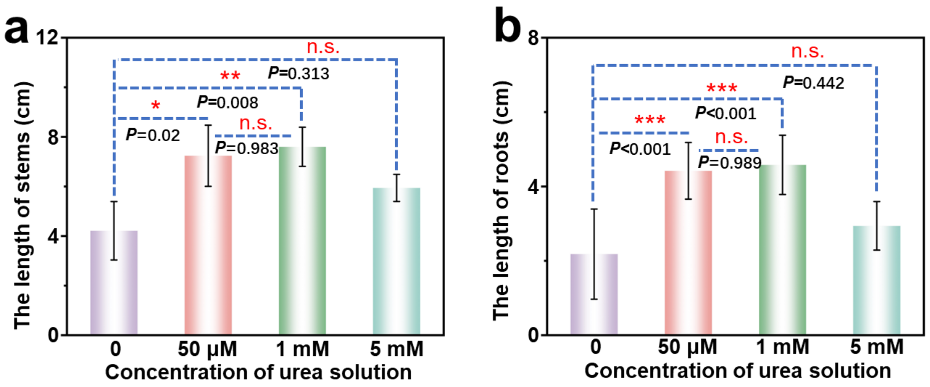

Figure S9. Significant difference analysis of the length of (a) stems and (b) roots in different concentration of urea solution. Values are means ± s.d. (n= 20 independent pea seeds were randomly selected from each parallel sample for analysis using a random number table method). Data are expressed as mean ± SD; n.s.: no significant difference, *p<0.05, **p<0.01, ***p<0.001.

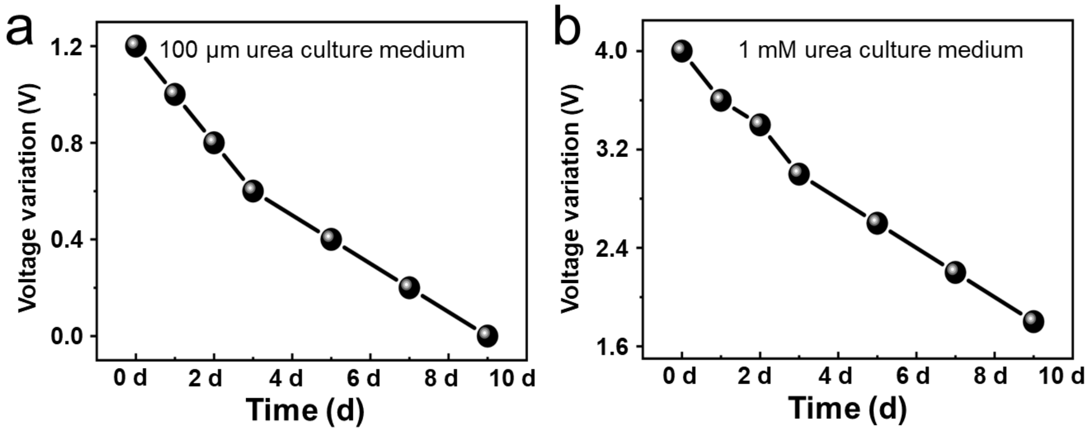

Figure S10. (a) Voltage variation of the self-powered urea sensor for analyzing culture medium with 100 μM urea after different incubation time. (b) Voltage variation of the self-powered urea sensor for analyzing culture medium with 1 mM urea after different incubation time.

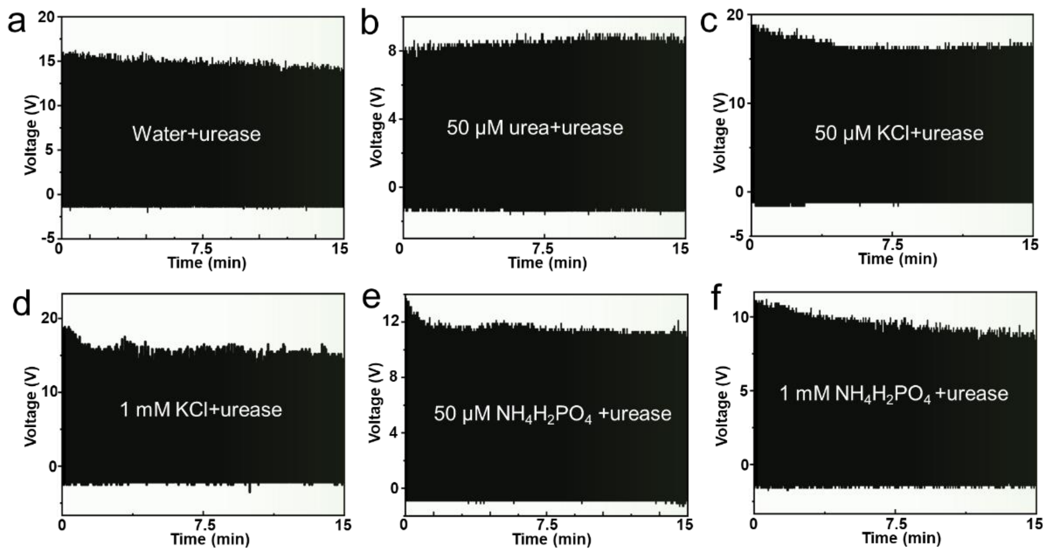

Figure S11. Change of device output voltage signals after adding urease.

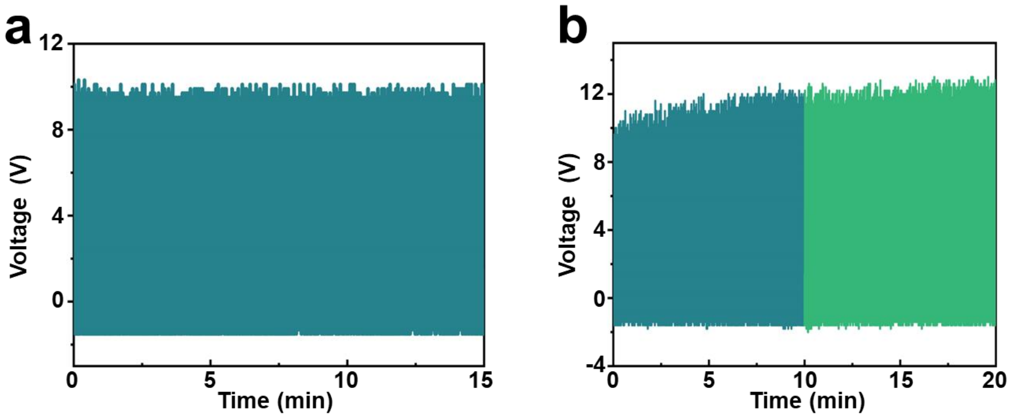

Figure S12. The variation of the output voltage signal of the device (a) before and (b) after adding urease into a mixed solution containing 500 μM urea, 500 μM potassium chloride, and 500 μM potassium dihydrogen phosphate.
